# GASTON-Mix: a unified model of spatial gradients and domains using spatial mixture-of-experts

**DOI:** 10.1093/bioinformatics/btaf254

**Published:** 2025-07-15

**Authors:** Uthsav Chitra, Shu Dan, Fenna Krienen, Benjamin J Raphael

**Affiliations:** Department of Computer Science, Princeton University, Princeon, NJ, 08540, United States; Eric and Wendy Schmidt Center, Broad Institute of MIT and Harvard, Cambridge, MA, 02142, United States; Princeton Neuroscience Institute, Princeton University, Princeton, NJ, 08540, United States; Princeton Neuroscience Institute, Princeton University, Princeton, NJ, 08540, United States; Department of Computer Science, Princeton University, Princeon, NJ, 08540, United States

## Abstract

**Motivation:**

Gene expression varies across a tissue due to both the organization of the tissue into *spatial domains*, i.e. discrete regions of a tissue with distinct cell type composition, and continuous *spatial gradients* of gene expression within different spatial domains. Spatially resolved transcriptomics (SRT) technologies provide high-throughput measurements of gene expression in a tissue slice, enabling the characterization of spatial gradients and domains. However, existing computational methods for quantifying spatial variation in gene expression either model only spatial domains—and do not account for continuous gradients of expression—or require restrictive geometric assumptions on the spatial domains and spatial gradients that do not hold for many complex tissues.

**Results:**

We introduce GASTON-Mix, a machine learning algorithm to identify *both* spatial domains and spatial gradients within each domain from SRT data. GASTON-Mix extends the *mixture-of-experts (MoE)* deep learning framework to a *spatial* MoE model, combining the clustering component of the MoE model with a *neural field* model that learns a separate 1D coordinate (“isodepth”) within each domain. The spatial MoE is capable of representing any geometric arrangement of spatial domains in a tissue, and the isodepth coordinates define continuous gradients of gene expression within each domain. We show using simulations and real data that GASTON-Mix identifies spatial domains and spatial gradients of gene expression more accurately than existing methods. GASTON-Mix reveals spatial gradients in the striatum and lateral septum that regulate complex social behavior, and GASTON-Mix reveals localized spatial gradients of hypoxia and TNF-α signaling in the tumor microenvironment.

**Availability and implementation:**

GASTON-Mix is available at https://github.com/raphael-group/GASTON-Mix.

## 1 Introduction

Spatially resolved transcriptomics (SRT) technologies measure the gene expression and spatial location of thousands of cells in a 2D tissue slice (Moffitt *et al.* 2018, Rodriques *et al.* 2019, Janesick *et al.* 2023). The recent development of these high-throughput SRT technologies has enabled the study of the spatial and molecular organization of many biological systems including the brain and tumor microenvironment (Hunter *et al.* 2021, Zhang *et al.* 2021).

Broadly, the major goal of spatial transcriptomic data analysis is to characterize spatial variation in gene expression in a tissue slice from SRT data. Gene expression varies spatially in a tissue due to both the organization of cell types as well as local gradations in cell state. Tissues are organized into spatial domains, or discrete regions of a tissue with distinct cell type composition and biological function. For example, the brain is divided into different regions consisting of different types of neurons, while tumors often contain multiple spatially coherent clones characterized by transcriptionally distinct subpopulations of cells. At the same time, many genes exhibit continuous spatial gradients of expression within a spatial domain. These spatial gene expression gradients are due to a variety of factors including cell type gradients within a domain, inter-cellular communication, and local changes in cell state and microenvironment. Gene expression gradients may be present even within cells of the same cell type. For instance, several genes exhibit spatial expression gradients within CA1 pyramidal neurons in the hippocampus (Cembrowski *et al.* 2016, Zeng 2022), while in a tumor clone, the expression of genes involved in metabolism or angiogenesis may vary continuously with the distance to the tumor boundary due to oxygen gradients or immune interactions (Smith and Hodges 2019).

A large number of computational methods have been introduced for identifying spatial domains from SRT data [e.g. described by Yuan *et al.* (2024)]. These methods typically assume that (mean) gene expression is constant within each domain, and that there are large, discontinuous changes in gene expression across domain boundaries. This assumption is explicitly made by hidden Markov random fields (HMRFs) (Dries *et al.* 2021, Zhao *et al.* 2021) and is implicitly made by algorithms which cluster cellular embedding vectors, e.g. methods which derive embeddings from graph neural networks (GNNs) (Hu *et al.* 2021, Dong and Zhang 2022, Long *et al.* 2023), variational autoencoders (VAEs) (Varrone *et al.* 2024), spatial kernels (Singhal *et al.* 2024), or cell type composition (Jackson *et al.* 2024). However, most existing methods for identifying spatial domains do not account for continuous gene expression gradients within a spatial domain; as we demonstrate in this work, not modeling continuous gradients may lead to inaccurate inference of spatial domains.

The standard approach for measuring spatial gradients of gene expression is to derive a 1D coordinate describing the relative position of a spatial location within a spatial domain, and estimate the rate of change of gene expression along this coordinate. Some methods attempt to derive such a 1D coordinate from low-dimensional embeddings obtained from matrix factorization or deep learning models (Ren *et al.* 2022, Chidester *et al.* 2023, Pham *et al.* 2023, Townes and Engelhardt 2023, Haviv *et al.* 2024), but because these methods do not explicitly model spatial gradients, the learned embeddings do not necessarily correspond to the relative position of a spatial location within a spatial domain. Further, the few existing approaches for explicitly identifying spatial expression gradients require substantial prior knowledge on the tissue geometry, e.g. manually annotated boundaries (Ma *et al.* 2022) or histological tumor annotations (Kueckelhaus *et al.* 2024), in order to estimate the relative position within a given spatial domain. Some of us recently introduced a method, GASTON, that learns a 1D coordinate called the isodepth that continuously varies across the entire tissue slice and can be used to quantify gene expression gradients within spatial domains (Chitra *et al.* 2025). The isodepth determines a “topographic map” of gene expression, but GASTON assumes that spatial domains are bounded by level sets of the isodepth coordinate. This restrictive geometric assumption often does not hold for complex tissues with an arbitrary arrangement of spatial domains; e.g. in this work we derive a tissue and gene expression function which we mathematically prove cannot be modeled by GASTON (Theorem 1). Thus, the problem of fully characterizing spatial variation in gene expression—by both identifying spatial domains and a 1D coordinate within each domain describing spatial gradients of expression—remains unsolved.

We introduce GASTON-Mix, an unsupervised method that simultaneously identifies spatial domains and derives gene expression gradients within spatial domains from SRT data. GASTON-Mix combines the sparsely-gated, mixture-of-experts (MoE) deep learning framework (Shazeer *et al.* 2017) with a neural field model (Xie *et al.* 2022) into a new model that we call a spatial MoE model. Sparsely-gated MoE models are widely used in large, transformer-based machine learning models such as ChatGPT due to their large model capacity and efficient training procedure (Shazeer *et al.* 2017), while neural field models are often used in computer graphics and vision for parametrizing physical properties of objects in space (Xie *et al.* 2022). GASTON-Mix uses the gating network of the sparsely-gated MoE model to represent any geometric arrangement of spatial domains in a tissue, and GASTON-Mix parametrizes the experts using a neural field model which learns a separate 1D isodepth coordinate and topographic map for each spatial domain. We show on simulated and real SRT data that GASTON-Mix more accurately identifies spatial domains and spatial gradients of gene expression compared to existing methods. On MERFISH data from the mouse brain, GASTON-Mix reveals spatial gradients in the striatum and lateral septum that regulate complex social behavior. On 10x Genomics Visium data from a breast cancer sample, GASTON-Mix reveals spatial gradients of hypoxia and TNF-α signaling that are localized to specific tumor domains.

## 2 Materials and methods

### 2.1 Gene expression functions and spatial gradients

We start by describing a general framework for spatial domains and gene expression gradients in SRT data following the exposition by Chitra *et al.* (2025). SRT technologies measure the expression of *G* genes in a tissue slice $T\subseteq R^{2}$, which we model using a (vector-valued) gene expression function $f:T\to R^{G}$. The vector $f(x,y)={\left(f_{1}(x,y),\ldots ,f_{G}(x,y)\right)}^{⊺}$ is the expression of genes g=1,…,G at spatial location (x,y) in the tissue slice *T*. The *g*th component function $f_{g}:R^{2}\to R$ describes the expression of a single gene *g*. For example, a gene *g* whose expression is constant across the tissue slice *T* has a constant expression function $f_{g}(x,y)=c$.

We model each gene expression function $f_{g}(x,y)$ as a piecewise, continuously differentiable function. Continuously differentiable functions, or functions *f* whose gradient ∇f is continuous, describe continuous gradients of gene expression (e.g. due to gradients in cell type proportion or cellular interactions), while piecewise functions allow for discontinuities in expression due to sharp changes in cell type composition or other factors. We assume the expression functions $f_{g}(x,y)$ have the same pieces for all genes g=1,…,G, and thus have the form


(1) $$f_{g}(x,y)=\sum_{p=1}^{P}f_{p,g}(x,y)\cdot 1_{\left\{(x,y)\in R_{p}\right\}},$$

where $R_{1},\ldots ,R_{P}\subseteq T$ is a partition of the tissue slice *T* into *P* disjoint regions, which we call spatial domains, and $f_{p,g}:R_{p}\to R$ is a continuously differentiable function describing the expression of gene *g* in domain $R_{p}$.

A spatial gradient describes how gene expression changes across the 2D tissue slice *T*. The spatial gradient for a single gene *g* at spatial location (x,y) is given by the gradient $\nabla f_{g}(x,y)$ of the expression function $f_{g}$. More generally, the rows of the Jacobian matrix $J(f)(x,y)={\left[\begin{array}{c} \nabla f_{1}(x,y)\;\cdots \;\nabla f_{G}(x,y) \end{array}\right]}^{⊺}\in R^{G\times 2}$ of the gene expression function f give the spatial gradient of each gene g=1,…,G at spatial location (x,y)∈T. Since the expression function $f_{g}$ is piecewise continuously differentiable, the spatial gradient $\nabla f_{g}$ is piecewise continuous and may be written is


(2) $$\nabla f_{g}(x,y)=\sum_{p=1}^{P}\nabla f_{p,g}(x,y)\cdot 1_{\left\{(x,y)\in R_{p}\right\}},$$

where $\nabla f_{p,g}$ is the gradient of the domain-*p* gene expression function $f_{p,g}$. We note that $\nabla f_{p,g}$ is a continuous function as $f_{p,g}$ is continuously differentiable.

### 2.2 GASTON framework and limitations

Directly estimating the spatial gradients $\nabla f_{g}$ for each gene g=1,…,G from SRT data is difficult due to the limited spatial resolution and/or sparsity of current SRT technologies. In our previous work, we proposed to estimate the spatial gradients $\nabla f_{g}$ by making several global assumptions on the structure of the spatial gradients $\nabla f_{g}$ and spatial domains $R_{p}$, implemented in the GASTON algorithm (Chitra *et al.* 2025). We briefly describe these assumptions.

First, GASTON assumes that there is a continuous vector field $v:T\to R^{2}$ such that the spatial gradient $\nabla f_{p,g}$ for each gene g=1,…,G in spatial domain p=1,…,P is proportional to the vector field v, i.e. $\nabla f_{p,g}(x,y)\propto v(x,y)$. In particular, GASTON assumes that the vector field v=∇d is the gradient of a continuously differentiable scalar function $d:R^{2}\to R$. The vector field v is called the spatial gradient vector field, and the scalar function d(x,y) is called the isodepth and describes the “topography” of the tissue slice *T*, analogous to the elevation in a topographic map of a geographic region. Second, GASTON assumes that each spatial domain $R_{p}$ is the union of level sets of the isodepth *d*.

GASTON uses these two assumptions to write the gene expression function f(x,y) as f(x,y)=h(d(x,y)), where $h(z):R\to R^{G}$ is a piecewise continuously differentiable function. GASTON approximates the functions d(x,y) and h(z) using neural networks. However, there exist biologically realistic expression functions f whose spatial domains $R_{p}$ cannot be described in such a way—and thus cannot be learned by GASTON. We formalize this claim by describing a simple and biologically plausible example gene expression function f(x,y) which we prove does not satisfy the GASTON assumptions.

**Gene expression function that cannot be modeled by single isodepth function used in GASTON.** Consider a rectangular tissue slice $T\subseteq R^{2}$ with P≥2 spatial domains where two domains $R_{1},R_{2}$ are adjacent squares. Suppose there exists a gene *g* with domain-1 and domain-2 expression functions $f_{1,g}(x,y),f_{2,g}(x,y)$, respectively, given by


(3) $$\begin{array}{c} f_{1,g}(x,y)=x\\ f_{2,g}(x,y)=y \end{array}$$

as shown in Fig. 1.

**Figure 1.**
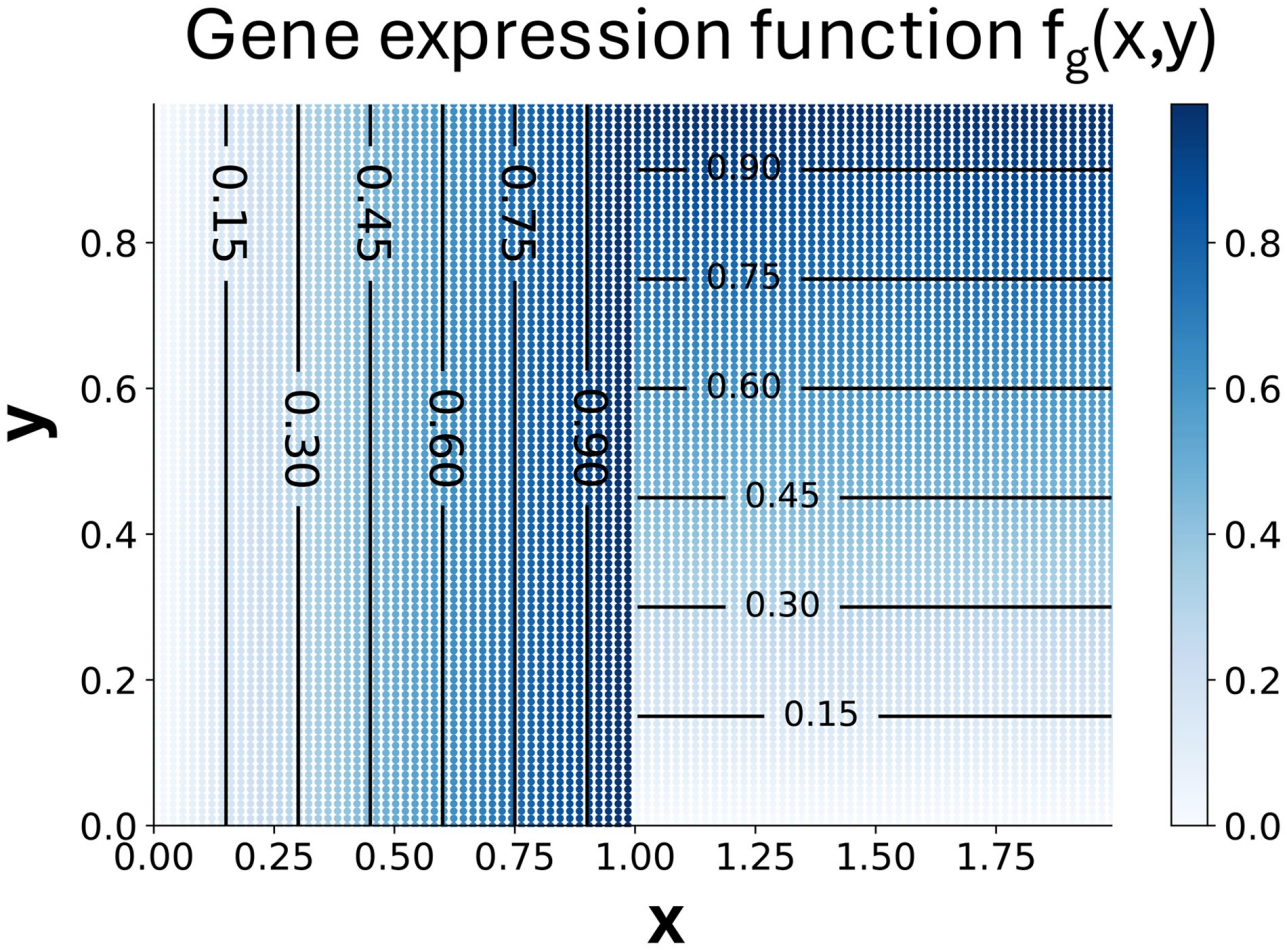
A piecewise continuous gene expression function $f_{g}(x,y)$ which cannot be described with a single, continuous isodepth function d(x,y) (Theorem 1).

We prove that the GASTON assumptions do not hold for this gene expression function f, as there do not exist a continuous function $d:R^{2}\to R$ and piecewise continuous function $h:R\to R^{G}$ such that f(x,y)=h(d(x,y)).

Theorem 1. *Let* $f:R^{2}\to R^{G}$  *satisfy (3) for some gene* g∈{1,…,G}*. There does not exist a continuously differentiable function* $d:R^{2}\to R$  *and piecewise continuously differentiable function* $h:R\to R^{G}$  *such that* f(x,y)=h(d(x,y)).

See the Appendix, available as supplementary data at *Bioinformatics* online, for a proof and Section 3.1 for an empirical demonstration of Theorem 1.

The fundamental reason that GASTON cannot model such a gene expression function f is because GASTON assumes that the function f has a global structure, in that there is a single, continuous isodepth function d(x,y) across the entire tissue *T* describing both the geometry of the spatial domains $R_{p}$ and the spatial gradients $\nabla f_{p,g}$ within each domain $R_{p}$. However, Theorem 1 shows that there are scenarios where the gene expression function f does not have such a structure. These theoretical and empirical observations motivate us to derive a model that relaxes GASTON’s global isodepth model.

### 2.3 Domain-specific spatial topography problem

We derive a model of spatial domains and local, domain-specific spatial gradients that relaxes the global assumptions of GASTON described in the previous section.

We start by describing a general model of spatial domains that formalizes the assumptions implicit in many spatial domain identification methods. We define the spatial domains $R_{p}$ in terms of an indicator function $w_{p}(x,y):R^{2}\to \{0,1\}$, where $w_{p}(x,y)=1$ if spatial location $(x,y)\in R_{p}$ and $w_{p}(x,y)=0$ otherwise. We call $w_{p}(x,y)$ the domain-*p* assignment function. Note that $\sum_{p=1}^{P}w_{p}(x,y)=1$ for every spatial location (x,y), as the spatial domains $R_{p}$ are disjoint. The gene expression function $f_{g}$ may be written as


(4) $$f_{g}(x,y)=\sum_{p=1}^{P}f_{p,g}(x,y)\cdot w_{p}(x,y).$$

We do not make any global assumptions on the structure of the domain-*p* assignment function $w_{p}(x,y)$, as the spatial domains of a tissue may have arbitrary shape and arrangement. In particular, unlike GASTON, we do not assume that the domain-*p* assignment function $w_{p}(x,y)$ is related to the contours of a continuous function d(x,y). We also relax GASTON’s assumption that there is a global spatial gradient vector field across all domains. Instead, we assume that *each* domain $R_{p}$ has its own continuous spatial gradient vector field $v_{p}:R_{p}\to R^{2}$. The spatial gradients ${\left(\nabla f_{p,g}(x,y)\right)}_{g=1}^{G}$ in domain-*p* for each gene g=1,…,G are proportional to $v_{p}(x,y)$; i.e. $\nabla f_{p,g}(x,y)=\beta_{p,g}\cdot v_{p}(x,y)$ for some constant $\beta_{p,g}\in R$.

We emphasize that we allow discontinuities in the domain-specific vector fields $v_{p}$ on the boundaries $\partial R_{p}$ of spatial domains $R_{p}$. That is, the tissue-wide vector field $v(x,y)=\sum_{p=1}^{P}v_{p}(x,y)\cdot w_{p}(x,y)$ formed by the piecewise sum of the domain-specific vector fields $v_{p}$ may be discontinuous. In contrast, GASTON’s global model does not allow for discontinuities on the boundaries $\partial R_{p}$ of domains $R_{p}$.

Further, we assume that each domain-*p* spatial gradient vector field $v_{p}$ is a conservative vector field, so that $v_{p}=\nabla d_{p}$ is the gradient of a continuously differentiable function $d_{p}:R_{p}\to R$ on the spatial domain $R_{p}$ which we call the domain-p isodepth function. Then the spatial gradients may be written as


(5) $$\nabla f_{p,g}(x,y)=\beta_{p,g}\cdot \nabla d_{p}(x,y).$$

Integrating both sides of (5) yields the following expression for the domain-*p* expression function $f_{p,g}$:


(6) $$\begin{array}{cc} & \nabla f_{p,g}(x,y)=\beta_{p,g}\cdot \nabla d_{p}(x,y)\\ & \Rightarrow f_{p,g}(x,y)=\beta_{p,g}\cdot d_{p}(x,y)+\alpha_{p,g}\overset{\text{def}}{=}h_{p,g}(d_{p}(x,y)) \end{array}$$

where $\alpha_{p,g},\beta_{p,g}\in R$ are constants and we define the linear function $h_{p,g}(z)=\beta_{p,g}z+\alpha_{p,g}$. That is, the domain-*p* expression function $f_{p,g}$ is the composition of the domain-*p* isodepth $d_{p}(x,y)$ and a linear function $h_{p,g}(z)$. We call $h_{p,g}(z)$ the domain-*p* one-dimensional (1D) expression function, as $h_{p,g}(z)$ is a function of a single variable *z* in contrast to the expression function $f_{p,g}(x,y)$ which is a function of two variables *x*, *y*. Then the gene expression function $f_{g}(x,y)$ across the entire tissue is given by


(7) $$\begin{array}{c} f_{g}(x,y)=\sum_{p=1}^{P}(\beta_{p,g}\cdot d_{p}(x,y)+\alpha_{p,g})\cdot w_{p}(x,y). \end{array}$$

Given SRT data and a number *P* of domains, we compute the maximum likelihood estimators (MLEs) of the spatial domain assignment functions $w_{p}(x,y)$, the domain-*p* isodepth functions $d_{p}(x,y)$, and the domain-*p* 1D expression functions $h_{p,g}(z)$. The observed SRT data consists of a transcript count matrix $A=[a_{i,g}]\in R^{N\times G}$, where $a_{i,g}$ is the expression of gene g∈{1,…,G} in spatial location i∈{1,…,N}, and a spatial location matrix $S=[s_{i}]\in R^{N\times 2}$, where $s_{i}=(x_{i},y_{i})$ is the *i*th observed spatial location. We define the domain-specific spatial topography problem (DS-STP) as the following maximum likelihood estimation problem.

**Domain-specific spatial topography problem (DS-STP).** *Given SRT data* (A,S)  *and a number P of spatial domains, find indicator functions* $w_{p}:R^{2}\to \{0,1\}$*, continuously differentiable functions* $d_{p}:R^{2}\to R$*, and linear functions* $h_{p,g}$  *for domains* p=1,…,P  *and genes* g=1,…,G  *that maximize the log-likelihood of the observed data:*


(8) $$\begin{array}{c} \underset{\begin{array}{c} \overset{w_{p}:R^{2}\to \{0,1\}\;s.t.\;}{\sum_{p=1}^{P}w_{p}(x,y)=1\forall (x,y)\in T}\\ d_{p}(x,y)\in C^{1}(R^{2},R)\\ h_{p,g}(z)\in L(R,R) \end{array}}{\text{arg}\;\text{max}}\;\;\sum_{g=1}^{G}\sum_{i=1}^{N}\;\text{log}\;P(a_{i,g}\sum_{p=1}^{P}|h_{p,g}(d_{p}(x_{i},y_{i}))n\\ \cdot w_{p}(x_{i},y_{i})) \end{array}$$

*where* $C^{1}(R^{2},R)$  *is the space of continuously differentiable functions from* $R^{2}$  *to* R  *and* L(R,R)  *is the space of linear functions from* R  *to* R.

The DS-STP problem considerably generalizes the spatial topography problem (STP) from Chitra *et al.* (2025). In the STP, one aims to learn a single continuous isodepth function d(x,y) for all domains, and assumes the spatial domain assignment functions $w_{p}(x,y)$ are level sets of the isodepth function *d*. In contrast, in the DS-STP, we aim to learn multiple isodepth functions $d_{p}$ and do not place any constraints on the spatial domain assignment functions $w_{p}(x,y)$.

The DS-STP is a computationally challenging problem to solve as it involves optimizing over spaces of both continuous and binary functions. Such an optimization problem is a generalization of mixed-integer optimization problems, where one optimizes over continuous and discrete variables (rather than functions). Since mixed-integer optimization problems are NP-hard (Karp 2010), it is unlikely that one would be able to solve the DS-STP to optimality.

### 2.4 Spatial MoE model

We introduce a spatial MoE model to efficiently and approximately solve the DS-STP. Our new model combines the widely used sparsely-gated, MoE deep learning framework with a neural field model.

Briefly, a sparsely-gated MoE model consists of *P* “expert” neural networks $E_{1},\ldots ,E_{P}$; a “gating” (or “routing”) neural network $G=(g_{1},\ldots ,g_{P})\in {\left[0,1\right]}^{P}$; and a fixed integer k>0. Each input s is mapped to a *sparse* linear combination $\sum_{p\in T_{k}(G(s))}g_{p}(s)\cdot E_{p}(s)$, where the linear coefficients $g_{p}(s)$ are given by the gating network G(s) evaluated on the input s, and the sum is taken over the set $T_{k}(G(s))\subseteq \{1,\ldots ,P\}$ of the *k* largest indices of the vector G(s); such a sum is sometimes called “top-*k* gating” in the deep learning literature (Shazeer *et al.* 2017). The expert networks $E_{1},\ldots ,E_{P}$ and gating network *G* are trained jointly using backpropagation.

**Figure 2.**
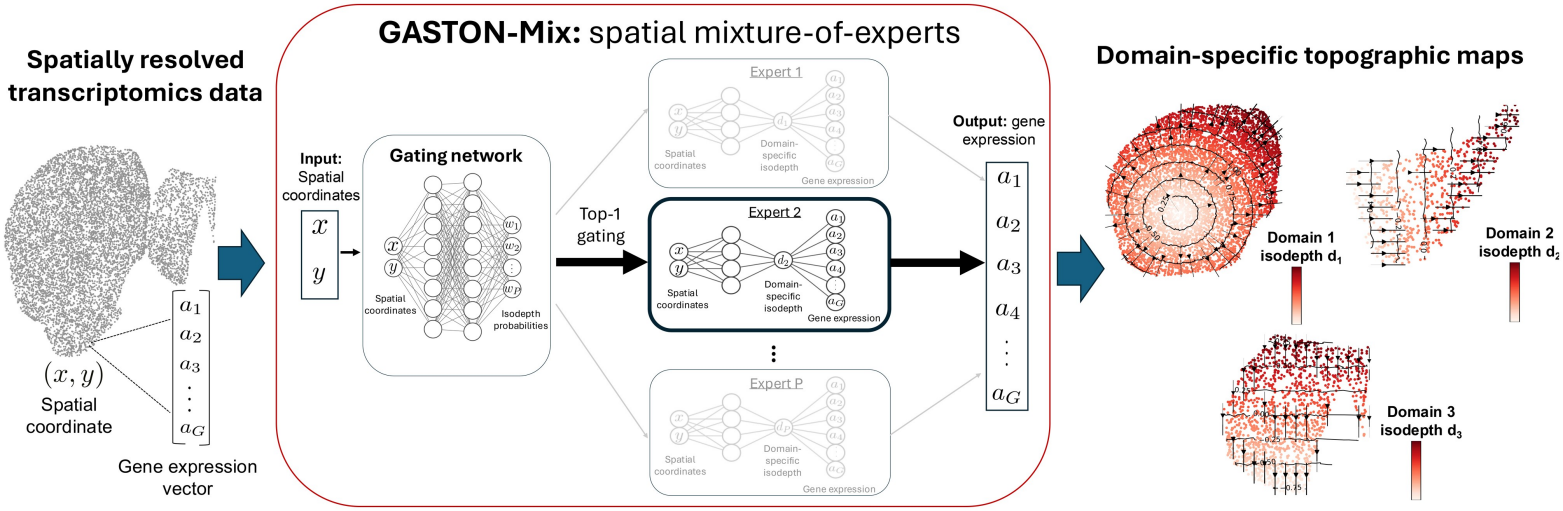
(Left) The input to GASTON-Mix is spatially resolved transcriptomics (SRT) data from a 2D tissue slice, which contains the spatial location (x,y) and gene expression $a=[a_{g}]\in R^{G}$ of thousands of cells in the tissue slice *T*. (Middle) GASTON-Mix is an unsupervised, spatial mixture-of-experts (MoE) deep learning model whose input is the spatial coordinate (x,y) of a cell and whose output is the predicted gene expression vector a. GASTON-Mix first uses a *gating network* $G(x,y)=(w_{1}(x,y),w_{2}(x,y),\ldots ,w_{P}(x,y))$ to compute a *soft assignment* of a cell to one of *P* experts, where each expert corresponds to a *spatial domain* $R_{p}$ of the tissue slice *T*. GASTON-Mix then uses top-1 gating to make a *hard assignment* of a cell to an expert network $E_{p}$. Each expert network $E_{p}(x,y)$ predicts gene expression from the spatial coordinate, and has a hidden layer of size 1 corresponding to the *isodepth* coordinate within each domain $R_{p}$. (Right) The domain-specific isodepth coordinate $d_{p}$ learned by each expert network $E_{p}$ describes a *topographic map* within each spatial domain $R_{p}$ and can be used to measure gradients of expression or cell type within the spatial domain.

We specifically use a sparsely-gated MoE model where the input s=(x,y) to the model is a spatial location (x,y) in the tissue slice *T* and the output is the predicted expression vector $a\in R^{G}$ at location (x,y). The *p*th expert $E_{p}:R^{2}\to R^{G}$ corresponds to the gene expression function ${\left(f_{p,g}(x,y)\right)}_{g=1}^{G}$ for domain-*p*, i.e.


(9) $$E_{p}(x,y)={\left(f_{p,g}(x,y)\right)}_{g=1}^{G}=(h_{p,g}{\left(d_{p}(x,y)\right)}_{g=1}^{G},$$

where the second equality uses (6). We parametrize the domain-*p* isodepth function $d_{p}(x,y)$ with a neural network as the universal approximation theorem shows that continuous functions can be closely approximated with neural networks (Hornik *et al.* 1989). Since $h_{p,g}(z)$ is a linear function, this is equivalent to parametrizing the *p*th expert $E_{p}$ with a neural network whose last hidden layer has a single node and is followed by a linear layer (Fig. 2).

Further, we use top-1 gating (i.e. k=1) so that each input spatial location s=(x,y) is assigned to a single expert $E_{p}$. Thus each expert ideally corresponds to an individual spatial domain $R_{p}$ in the tissue. We parametrize the gating function $G(x,y)=(g_{1}(x,y),\ldots ,g_{P}(x,y)):R^{2}\to R^{P}$ with a neural network whose last layer is a softmax, so that the entries ${\left(g_{p}(x,y)\right)}_{p=1}^{P}$ of the gating network are positive and sum to 1, i.e. $g_{p}(x,y)>0$ and $\sum_{p=1}^{P}g_{p}(x,y)=1$. In this way, the gating network $G(x,y)=(g_{1}(x,y),\ldots ,g_{P}(x,y)):R^{2}\to R^{P}$ makes a *soft* assignment of spatial location s=(x,y) to the *P* spatial domains. Moreover, since we use top-1 gating, the output of our model is the q(x,y)th expert $E_{q(x,y)}(x,y)$, where $q(x,y)=\text{arg}\;{\text{max}}_{p=1,\ldots ,P}(g_{1}(x,y),\ldots ,g_{P}(x,y))$ is the largest index in the entries of the gating network output G(x,y) (in the case of a tie, the smallest index with the largest entry returned). That is, the output $E_{q(x,y)}(x,y)$ corresponds to a hard assignment of each spatial location s=(x,y) to the q(x,y)th spatial domain.

We call our model a spatial MoE model as we use neural networks whose inputs are spatial coordinates to parametrize the our expert neural networks $E_{p}(x,y)$ and the gating neural network G(x,y) of an MoE model. Neural networks whose inputs are spatial coordinates are typically called neural field models or spatial implicit neural representations in the machine learning literature (Xie *et al.* 2022, Cole *et al.* 2023). To the best of our knowledge, this work and our previous work (Chitra *et al.* 2025) are the only papers that fit neural field models to spatial transcriptomics data. We note that a recent machine learning paper (Dryden and Hoefler 2022) also proposed a spatial MoE model for weather prediction and forecasting. However, their model differs from ours in two important ways: (1) their gating network does not use spatial information, and so their model does not identify spatial domains and (2) their expert networks do not learn an interpretable 1D coordinate for measuring spatial gradients.

### 2.5 GASTON-Mix

We implement our spatial MoE model in a package called GASTON-Mix (Fig. 2). GASTON-Mix is available at https://github.com/raphael-group/GASTON-Mix with the following implementation choices.

**Probability model and input features.** The MoE model described above can be implemented with different probability distributions for the observed gene expression values $a_{i,g}$. Following prior work (Townes *et al.* 2019, Sarkar and Stephens, 2021), we model the UMI counts $a_{i,g}$ with a Poisson distribution of the form $a_{i,g}\overset{i.i.d.}{∼}\text{Pois}\left(U_{i}\cdot \;\text{exp}(f_{g}(x_{i},y_{i}))\right)$ where $U_{i}$ is the total UMI count at spatial location $\left(x_{i},y_{i}\right)$. In practice, while one could directly solve the DS-STP with all or selected UMI counts, for efficiency we do not directly solve the DS-STP in this way. Instead, we solve the DS-STP using the top generalized linear model principal components (GLM-PCs) (Townes *et al.* 2019) under a Gaussian error model. We then use the estimated isodepth functions ${\hat{d}}_{p}$ and spatial domain assignment functions ${\hat{w}}_{p}$ to estimate the 1D gene expression functions ${\hat{h}}_{p,g}$ for each gene g=1,…,G by solving G·P Poisson regression problems, following Chitra *et al.* (2025). See Appendix, available as supplementary data at *Bioinformatics* online, for details.

**Neural network architecture and training.** We parametrize the gating network $G:R^{2}\to R^{P}$ with a neural network with two hidden layers of size 20, and we parametrize the domain-*p* isodepth function $d_{p}:R^{2}\to R$ with a neural network with one hidden layer of size 20. We empirically observe that such an architecture is able to represent the arrangement of spatial domains in most tissues as well as the isodepth within each spatial domain without overfitting to the data. We implement GASTON-Mix in PyTorch. We train GASTON-Mix for 50 000 epochs using a full batch; training takes roughly 10 min for the datasets shown in the manuscript with a single P100 GPU. We use an alternating maximization approach, where we alternate between updating the gating function *G* and the experts $E_{1},\ldots ,E_{P}$, as we empirically observe that an alternating optimization approach leads to a lower loss (i.e. larger likelihood (8)). In contrast to GASTON which requires training 30 separate models with different random initializations, we train our spatial MoE model once with a single initialization.

**Initialization and model selection.** We often have prior knowledge in the form of spatial domain labels $\ell_{i}\in \{1,\ldots ,P\}$ for each spatial location i=1,…,N, e.g. from a clustering algorithm such as *k*-means clustering. While these spatial domain labels $\ell_{i}$ are likely incorrect, it may still be useful to incorporate such prior knowledge into our model and allow the model to “refine” the noisy domain labels during training. In such cases, we first initialize the gating network G(x,y) by training the gating network *G* to predict the label $\ell_{i}$ of each spatial location $\left(x_{i},y_{i}\right)$ using a multi-class cross-entropy classification loss (Bishop and Nasrabadi 2006). We use the labels from *k*-means clustering (Bishop and Nasrabadi 2006) in our simulations (Section 3.1) and the labels from CellCharter (Varrone *et al.* 2024) in our real data evaluation (Section 3.2). See the Appendix, available as supplementary data at *Bioinformatics* online, for a quantitative evaluation of our initialization. We set the number *P* of experts equal to either the ground truth number of domains if known (e.g. in simulations) or the number of clusters determined by CellCharter.

**Identifying gene expression gradients.** A large (absolute) value of the slope $\beta_{p,g}$ of the 1D gene expression function $h_{p,g}(z)$ indicates a continuous gradient in expression for gene *g* in domain $R_{p}$. Following Chitra *et al.* (2025), we say that gene *g* exhibits an expression gradient in domain $R_{p}$ if the absolute slope $\left|\beta_{p,g}\right|$ is greater than a threshold $s_{p}$. We set $s_{p}$ to be the fifth percentile of all estimated slope magnitudes ${\left(\left|\beta_{p,g}\right|\right)}_{g=1,\ldots ,G}$ in domain $R_{p}$.

**Cell type-specific gradients.** Gradients in gene expression, i.e. large absolute values of the slope $\beta_{p,g}$, may result from spatial variation in cell type proportion. For single-cell data with known cell type annotations, we follow our earlier approach (Chitra *et al.* 2025) to distinguish between cell type-specific gradients and gradients due to other variation; see Appendix, available as supplementary data at *Bioinformatics* online.

## 3 Results

### 3.1 Evaluation on simulated SRT data

We first evaluated GASTON-Mix on simulated SRT data. We compared GASTON-Mix to four recent methods for analyzing SRT data: BANKSY (Singhal *et al.* 2024), CellCharter (Varrone *et al.* 2024), GraphST (Long *et al.* 2023), and GASTON (Chitra *et al.* 2025). BANKSY, CellCharter, and GraphST are methods for spatial domain identification which do not explicitly model gene expression gradients within domains, while GASTON is primarily designed for identifying spatial gradients and makes restrictive global assumptions on tissue geometry.

**Figure 3.**
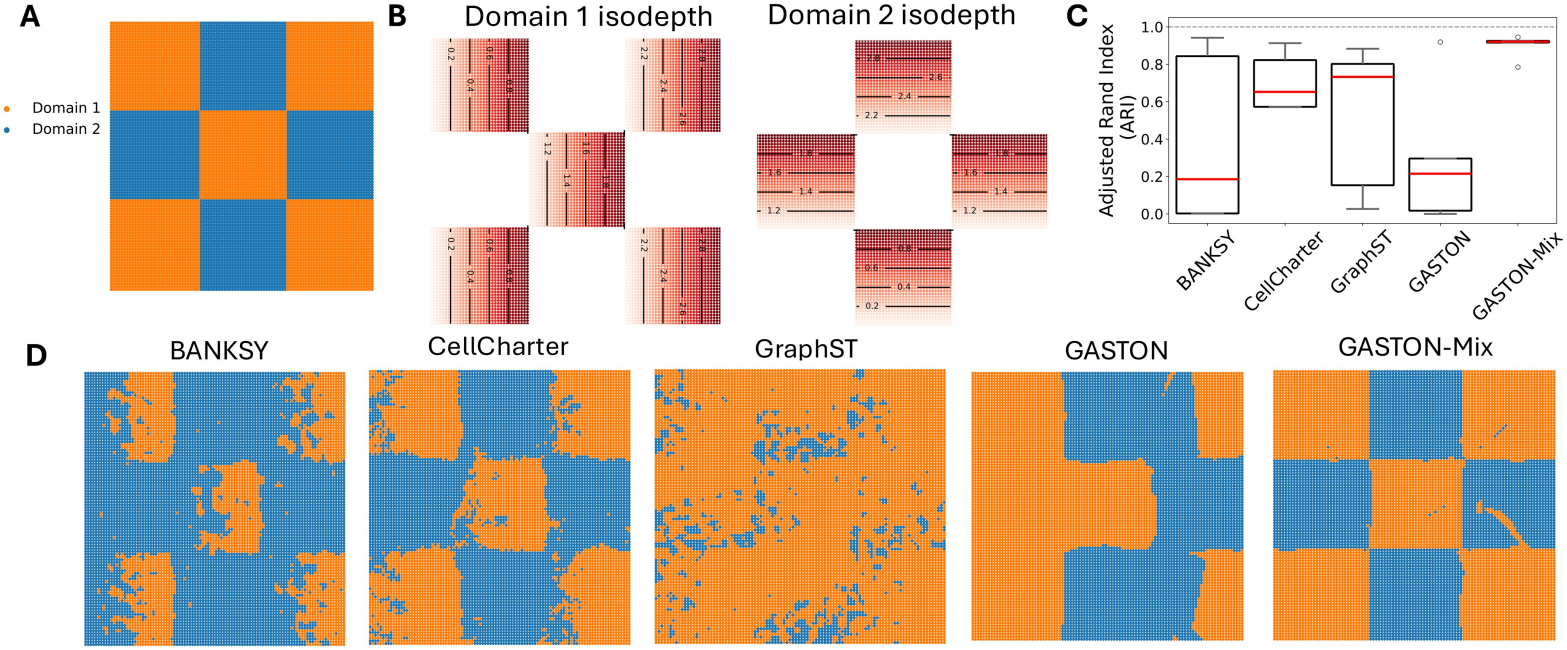
(A) Spatial domains of simulated SRT dataset in a rectangular tissue $T=R_{1}\cup R_{2}$ with two domains $R_{1}$ and $R_{2}$ arranged in a “checkerboard” pattern. (B) Domain-specific isodepth functions $d_{1}(x,y)$ and $d_{2}(x,y)$ for domains 1 (left) and 2 (right), respectively. Lines denote contours of constant isodepth. (C) Adjusted Rand index (ARI) of spatial domains identified by BANKSY (Singhal *et al.* 2024), CellCharter (Varrone *et al.* 2024), GraphST (Long *et al.* 2023), GASTON (Chitra *et al.* 2025), and GASTON-Mix over five simulated instances. Red lines indicate median ARI. (D) Spatial domains identified by each method for a single simulated instance.

We simulated SRT data (S,A) on a rectangular tissue *T* with N=10 000 spatial locations consisting of P=2 spatial domains $R_{1},R_{2}$ arranged in a “checkerboard” pattern (Fig. 3A), where the domain-1 isodepth function $d_{1}(x,y)$ (resp. domain-2 isodepth $d_{2}(x,y)$) is the distance from the nearest left edge (resp. bottom edge) of a square in the checkerboard (Fig. 3B). We simulate the expression of G=15 genes using gene expression functions $f_{g}(x,y)$ that satisfy the assumptions of Theorem 1; see Appendix, available as supplementary data at *Bioinformatics* online for details.

We find that GASTON-Mix achieves much higher accuracy in spatial domain identification, measured with the adjusted Rand index (ARI), compared to BANKSY, CellCharter, GraphST, or GASTON (GASTON-Mix mean ARI ≈0.85 versus mean ARI <0.70 for other methods; Fig. 3C). The lower ARI of BANKSY, GraphST, and CellCharter is likely because these methods do not explicitly model gene expression gradients, while GASTON’s poor performance is because the simulated data does not satisfy GASTON’s global isodepth assumption (Theorem 1). While BANKSY claims to model “gradients in gene expression in [cellular] neighborhoods” (Singhal *et al.* 2024), the BANKSY domains have the lowest ARI. In contrast, GASTON-Mix’s local model of tissue geometry and expression gradients allows for accurate inference of spatial domains.

**Figure 4.**
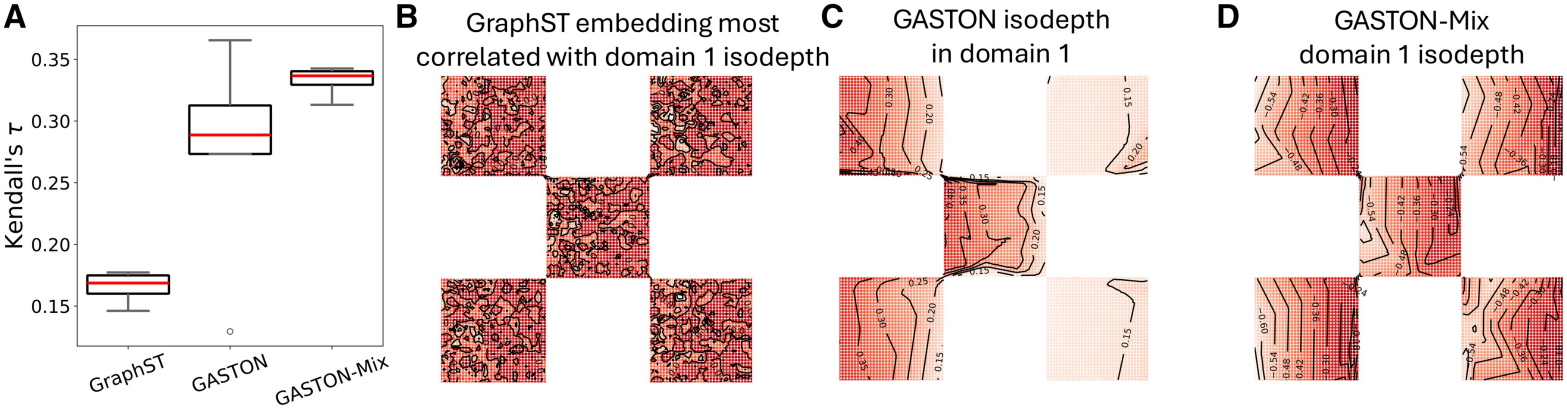
(A) Kendall’s τ coefficient between simulated domain-specific isodepth (shown in 3B) and the estimated isodepth from GraphST, GASTON, and GASTON-Mix. (B) GraphST embedding most correlated with the true isodepth, (C) GASTON isodepth, and (D) GASTON-Mix domain-1 isodepth in the true domain 1. Curves denote contours of constant value.

We next evaluated the ability of GASTON-Mix and other methods to learn spatial gradients. We specifically evaluated how well each method learns the domain-specific isodepth functions $d_{i}(x,y)$, which provide coordinates for quantifying spatial gradients. We find (Fig. 4A) that GASTON-Mix has larger Kendall’s τ correlation (τ≈0.33) with the true domain-specific isodepths than GASTON (τ≈0.27) or GraphST (τ≈0.17). GraphST is unable to learn a spatially smooth embedding (Fig. 4B) while the GASTON isodepth function is constant in several of the squares (Fig. 4C), likely because there does not exist a continuous isodepth function *d* that describes the gene expression function f(x,y) (Theorem 1). In contrast, the GASTON-Mix domain-specific isodepths smoothly vary across the domains (Fig. 4D), showing the advantages of learning local topographic maps.

### 3.2 Analysis of mammalian forebrain structures

**Figure 5.**
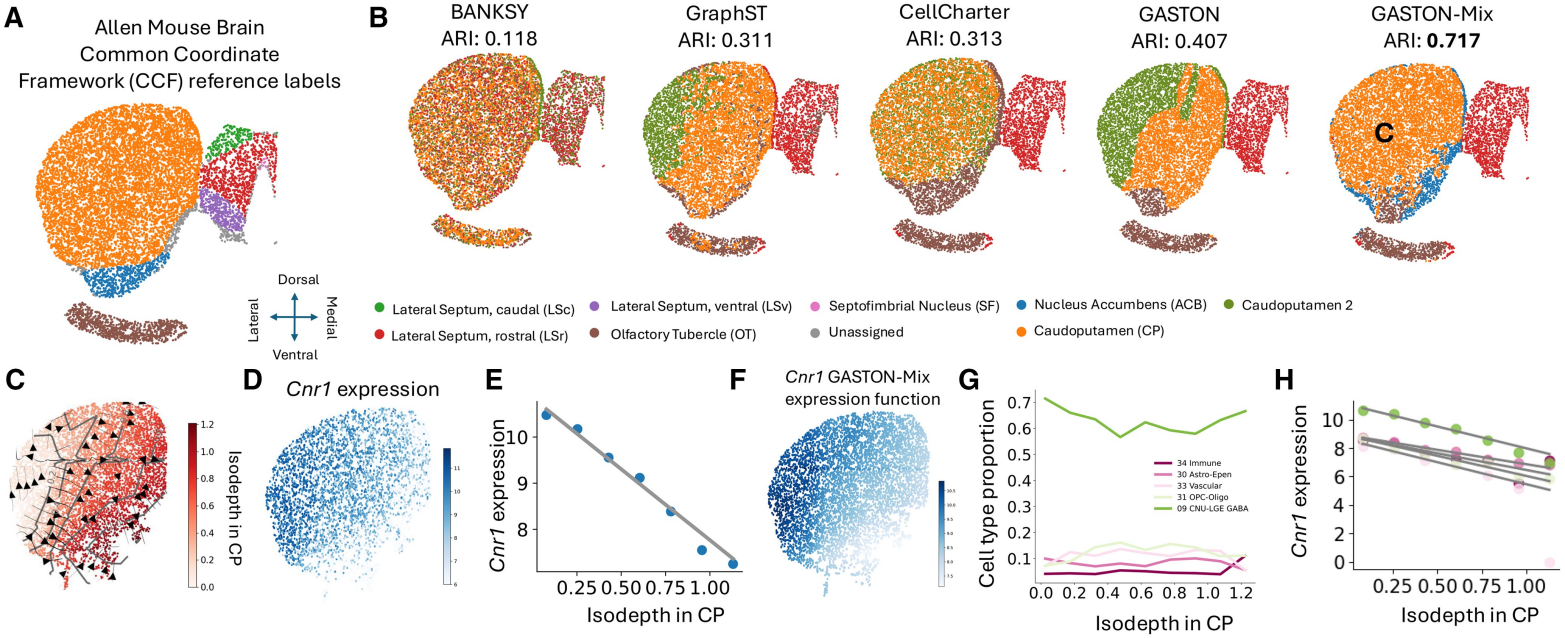
(A) Anatomical labels of each cell in a MERFISH mouse anterior forebrain sample from the Allen Mouse Brain Common Coordinate Framework (CCF) (Zhang *et al.* 2021). (B) Spatial domains and adjusted Rand index (ARI) compared to CCF labels in (A) for five different methods: BANKSY (Singhal *et al.* 2024), GraphST (Long *et al.* 2023), CellCharter (Varrone *et al.* 2024), GASTON (Chitra *et al.* 2025), and GASTON-Mix. (C) GASTON-Mix isodepth ${\hat{d}}_{p,g}$ for GASTON-Mix-inferred caudoputamen (CP, i.e. the orange region labeled with C in (B)). (D) *Cnr1* expression shown in log counts per million (CPM). (E) *Cnr1* expression versus CP-specific isodepth ${\hat{d}}_{p,g}$ learned by GASTON-Mix. (F) GASTON-Mix *Cnr1* expression function ${\hat{f}}_{p,g}$ in CP domain. (G) Cell type proportion versus CP-specific isodepth ${\hat{d}}_{p,g}$ for five most prevalent cell types. (H) GASTON-Mix cell type-specific *Cnr1* expression functions ${\hat{h}}_{c,p,g}$ colored by cell types in (G).

We next evaluated GASTON-Mix on a sample of anterior structures of the mouse forebrain, where MERFISH was applied to measure the expression of 1, 122 genes in 9696 cells (Zhang *et al.* 2021). This data is highly sparse, with a median of 312 measured transcripts per cell. The mouse forebrain has a complex geometry, consisting of several spatial domains of differing sizes and relative positions including the striatum (consisting of the dorsal caudoputamen and ventrally located nucleus accumbens), olfactory tubercle, and lateral septum, as labeled in the Allen Mouse Brain Common Coordinate Framework (CCF, Fig. 5A) (Wang *et al.* 2020). Furthermore, earlier prominent biological studies have reported several gradients of gene expression, neurochemicals, and connectivity across different regions of the striatum and lateral septum and in different directions, including the dorsolateral–ventromedial gradient in the striatum (Kawagoe *et al.* 1992, Voorn *et al.* 2004) and the dorsal–ventral gradient in the lateral septum (Reid *et al.* 2024) (Fig. 5A). Thus, we hypothesized that this MERFISH sample would pose a challenge for existing spatial domain identification methods that do not account for such gradients, but also for algorithms like GASTON which make global, geometric assumptions about tissue geometry.

We compared the spatial domains identified by GASTON-Mix to the domains identified by BANKSY, CellCharter, GraphST, and GASTON. We find that GASTON-Mix has larger agreement with the CCF domain labels, measured by the adjusted Rand index (ARI, Fig. 5B), compared to the four other methods (GASTON-Mix ARI =0.717 versus ARI <0.41 for other methods). In particular, GASTON-Mix is the only method which resolves the caudoputamen (CP, orange domain in Fig. 5A) as a single domain while other methods split this domain into two or more domains, with the Banksy and CellCharter output showing little spatial coherence in the orange domain. The reason the other methods fail to identify the CP domain is likely because of the prominent dorsolateral–ventromedial gradient in the domain (Kawagoe *et al.* 1992, Voorn *et al.* 2004, Bonev *et al.* 2024). We also note that GASTON, a method primarily developed to identify spatial gradients, has a larger ARI than the other methods which are specialized for spatial domain identification (GASTON ARI =0.407 versus BANKSY, GraphST, CellCharter ARI <0.32, Fig. 5B), demonstrating the difficulty that existing domain identification methods have when the data contains large gene expression gradients.

The domain-specific isodepth functions ${\hat{d}}_{p,g}$ learned by GASTON-Mix reveal several biologically relevant expression gradients that are not readily apparent from the raw MERFISH data. In particular, GASTON-Mix learns a domain-specific isodepth $d_{p,g}$ that smoothly varies across the domain in the GASTON-Mix-inferred caudoputamen (CP, Fig. 5C), allowing for the identification of genes *g* with CP-specific expression gradients, i.e. with large absolute slopes $\left|\beta_{p,g}\right|$. Such a coordinate is not learned by SRT embedding methods which learn spatially incoherent embeddings, e.g. the GraphST embeddings in the CP domain (Fig. S2, available as supplementary data at *Bioinformatics* online). GASTON-Mix identifies *Cnr1—*a reported marker for the dorsolateral–ventromedial striatum gradient (Märtin *et al.* 2019, He *et al.* 2021)—as having a CP-specific expression gradient (see Section 2). *Cnr1* has sparse expression in the domain with a median expression of 1 per cell (Fig. 5D). GASTON-Mix aggregates expression across contours of constant isodepth (Fig. 5C) and learns a 1D linear gene expression function $h_{p,g}(z)$ (Fig. 5E) and a 2D expression function $f_{p,g}(x,y)=h_{p,g}(d_{p}(x,y))$ (Fig. 5J) that more readily displays the continuous expression gradients compared to the sparse expression. We also find that there are no large gradients of cell type proportion in the CP [Fig. 5G; cell type annotations from Zhang *et al.* (2021)] and that *Cnr1* exhibits a cell type-specific expression gradient across all cell types in the CP domain (see Section 2). This suggests that the *Cnr1* expression gradient is not driven by variation in cell type proportion and is likely due to other biological causes, e.g. gradients in excitatory input Voorn *et al.* (2004). We also observe similar patterns for other candidate markers for the dorsolateral–ventromedial striatum gradient in different species (Fig. S3, available as supplementary data at *Bioinformatics* online). Further, GASTON-Mix identifies biologically relevant spatial gradients in the lateral septum domain; such gradients have previously been observed in other spatial imaging data and are hypothesized to be involved in complex social behaviors (Risold and Swanson 1997, Chen *et al.* 2024, Reid *et al.* 2024). See Appendix and Fig. S4, available as supplementary data at *Bioinformatics* online, for details.

Overall, our results demonstrate that while existing SRT algorithms are challenged by spatial gradients, GASTON-Mix’s spatial MoE framework accurately identifies spatial domains and biologically relevant spatial gradients of gene expression.

### 3.3 Gradients in breast cancer tumor

**Figure 6.**
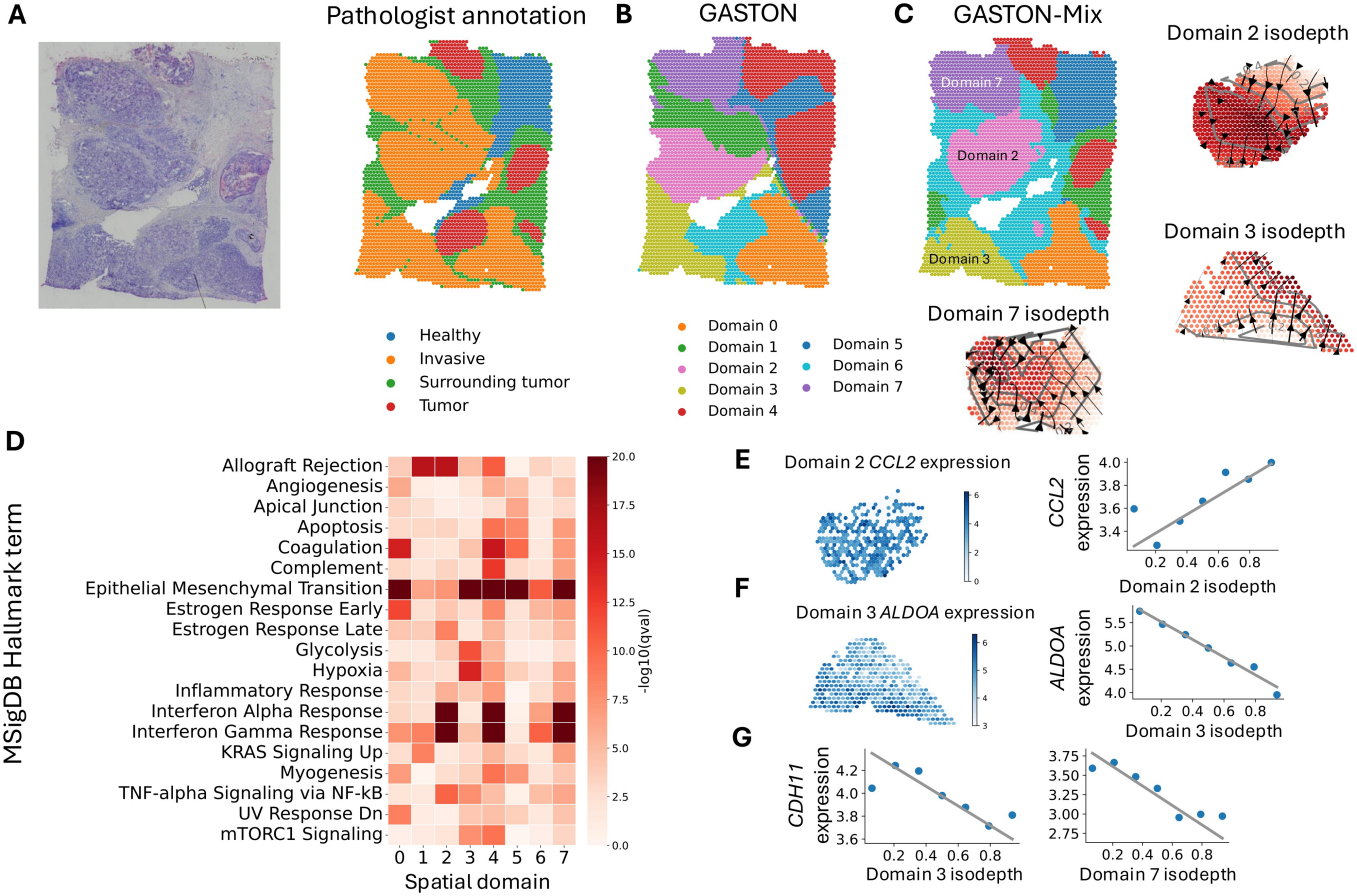
(A) (Left) H&E stain and (Right) pathologist annotation (Xu *et al.* 2022) for each spot (right) in a 10x Genomics Visium breast cancer sample. (B) Spatial domains identified by GASTON (Chitra *et al.* 2025). (C) Spatial domains identified by GASTON-Mix and inferred domain-specific isodepth functions $d_{p}$ for domains p=2,3,7. (D) Enrichment for hallmark cancer gene sets reported by gene set enrichment analysis (GSEA) (Kuleshov *et al.* 2016, FDR $<10^{-5}$) for genes with large expression gradients in each of the P=8 domains identified by GASTON-Mix. (E, F) (Left) Expression and (Right) Isodepth versus expression for (E) *CCL2* in domain 2 and (F) *ALDOA* in domain 3. (G) *CDH11* expression versus (Left) domain 3 isodepth and (Right) domain 7 isodepth.

We applied GASTON-Mix to a 10x Genomics Visium SRT sample of a human breast cancer (invasive ductal carcinoma; Fig. 6A). Physical and biochemical gradients within a tumor have been previously shown to promote cancer progression and metastasis (Oudin and Weaver 2016), but GASTON does not accurately identify the spatial domains in the tumor (Fig. 6B). GASTON-Mix accurately learns the domains of the tumor sample (Fig. 6C) and identifies several spatial gradient patterns of hypoxia and TNF-α signaling that are localized to specific tumor domains (Fig. 6D–G). See Appendix, available as supplementary data at *Bioinformatics* online, for details.

## 4 Discussion

We introduce GASTON-Mix, a deep learning algorithm for jointly learning spatial domains and gradients of gene expression within each spatial domain from SRT data. We derive a novel spatial MoE model which combines spatial clustering from the standard MoE model with a spatial neural field model that learns a 1D coordinate—the isodepth*—*within each spatial domain and gives the gradient direction of the expression of individual genes. Our spatial MoE model is capable of representing arbitrary geometric arrangements of spatial domains in a tissue, while the isodepth coordinates within each spatial domain describe domain-specific topographic maps that generalize the global topographic maps learned in our previous method GASTON (Chitra *et al.* 2025).

We anticipate many future directions for our work. These include: developing a statistical testing framework for whether a domain contains gene expression gradients; further studying the biological causes of the gene expression gradients identified by GASTON-Mix, e.g. by identifying cell–cell interactions that mediate expression gradients (Bafna *et al.* 2023, Sarkar *et al.* 2024); relaxing the GASTON-Mix assumptions to allow for inference of multiple gradient directions within a spatial domain as well as the optimal number of such gradient directions; and validating GASTON-Mix on tissues with more complex geometries, e.g. radial geometries. Ultimately, we believe that GASTON-Mix’s unified model of spatial domains and gradients provides a general framework for characterizing spatial variation in gene expression across complex tissues.

## Supplementary Material

btaf254_Supplementary_Data

## Data Availability

The GASTON-Mix software is available at https://github.com/raphael-group/GASTON-Mix/. The data underlying this article are available in the Brain Image Library at https://doi.org/10.35077/g.21 and in the 10x Genomics dataset repository at https://www.10xgenomics.com/products/spatial-gene-expression.
