# Supplementary material for: GASTON-Mix: a unified model of spatial gradients and domains using spatial mixture-of-experts
Source: Bioinformatics. 2025 Jul 15;41(Suppl 1):i523–32. doi: 10.1093/bioinformatics/btaf254 (PMC12261403; doi:10.1093/bioinformatics/btaf254)
Supplement: btaf254_Supplementary_Data [file btaf254_supplementary_data.pdf]

Appendix

Proof of Theorem 1

Without loss of generality, we assume the domains R_1 and R_2 are squares of side-length 1 that are side-by-side, where the bottom-left corner of square R_1 is $(0,0)$ and the bottom-left corner of square R_2 is $(1,0)$, as depicted in Figure 1.

We first prove the following lemma.

Lemma 1 Let $\mathbf{f}:\mathbb{R}^2\to\mathbb{R}^G$ be a function such that $\mathbf{f}(x,y)=\mathbf{h}(d(x,y))$ for continuous function $d:\mathbb{R}^2\to\mathbb{R}$ and piecewise continuous function $\mathbf{h}:\mathbb{R}\to\mathbb{R}^G$, and suppose there exists $g\in\{1,\dots,G\}$ such that f_g has the form in (3). Then for any $x_0\in[0,1]$, the function $e(y)=d(x_0,y):[0,1]\to\mathbb{R}$ is constant. Moreover, for any $x\in[1,2]$ and $y_0\in[0,1]$, the function $e'(x)=d(x,y_0)$ is constant.

Proof The second statement follows from a similar argument as the first statement, so we prove the first statement. Assume for the sake of contradiction that the first statement is not true. Then there exists $x_0\in[0,1]$ and $0\le y<y'\le 1$ such that $d(x_0,y)\neq d(x_0,y')$. Without loss of generality we may assume that $x_0=0$, and moreover that $d(0,y)<d(0,y')$ (e.g. by negating d).

Let $0<\varepsilon<d(0,y')-d(0,y)$ be sufficiently small. By Lemma 1, we have that $d(0,y)\neq d(x',y)$ for any $x'>0$. By continuity of d in R_1, there exists an $x'>0$ such that $d(x',y)=d(0,y)+\varepsilon$. Furthermore, by the intermediate value theorem for the univariate function $y\mapsto d(0,y)$, there exists a $y''\in(y,y')$ such that $d(0,y'')=d(0,y)+\varepsilon$.

Thus, we have that $d(x',y)=d(0,y'')=d(0,y)+\varepsilon$, which implies

$$x'=f_g(x',y)=h_g(d(x',y))=h_g(d(0,y''))=f_g(0,y'')=0, \tag{10}$$

where the first and last equalities follow by definition of f_g, the second and second-to-last equalities follow by definition of $\mathbf{f}$, and the third equality follows by definition of x' and y''. However this contradicts our assumption that $x'>0$. □

We now proceed to the proof of the Theorem.

Proof of Theorem 1 Assume for the sake of contradiction that there exists a continuous function $d:\mathbb{R}^2\to\mathbb{R}$ and piecewise continuous function $\mathbf{h}:\mathbb{R}\to\mathbb{R}^G$ such that $\mathbf{f}:\mathbb{R}^2\to\mathbb{R}^G$ can be written as $\mathbf{f}(x,y)=\mathbf{h}(d(x,y))$ for all $(x,y)\in T$.

From Lemma 1, we have that $d(x,y)$ is a function of the x-coordinate in R_1 and is a function of the y-coordinate in R_2. Thus, we may write $d(x,y)=a(x)\cdot 1_{\{(x,y)\in R_1\}}+b(y)\cdot 1_{\{(x,y)\in R_2\}}$ for continuous functions $a(x),b(y):\mathbb{R}\to\mathbb{R}$. Now, on the line $x=1$ where the squares overlap, then $d(1,y)|=a(1)$. Thus, $d(1,y)$ is constant for all $y\in[0,1]$. But by Lemma 1, for any $0\le y<y'\le 1$, we have that $d(1+\varepsilon,y)\neq d(1+\varepsilon,y')$; taking the limit as $\varepsilon\to 0$ implies that $d(1,y)\neq d(1,y')$ which is a contradiction. □

GASTON-Mix implementation

We solve the DS-STP using the top generalized linear model principal components (GLM-PCs) (Townes et al., 2019) under a Gaussian error model. We then use the estimated isodepth functions $\widehat{d}_p$ and spatial domain assignment functions $\widehat{w}_p$ to estimate the 1-D gene expression functions $\widehat{h}_{p,g}$ – and thus estimate the spatial domains $\widehat{R}_p$ – for each gene $g=1,\dots,G$ by solving $G\cdot P$ Poisson regression problems, following the procedure of Chitra et al. (2025). Our use of GLM-PCA is justified by the following observation from Ma et al. (2022): for SRT data $(\mathbf{A},\mathbf{S})$ where the UMI counts $a_{i,g}$ follow a Poisson distribution with mean $f_g(x_i,y_i)$ defined by (7), then the top-$2P$ GLM-PCs are approximately piecewise linear with Gaussian noise.

Specifically, given SRT data $(\mathbf{A},\mathbf{S})$, we first compute the top-$2P$ GLM-PCs $\mathbf{u}_j=[u_{i,j}]\in\mathbb{R}^N$ for $j=1,\dots,2P$. We then use the algorithm described in Section 2.4 to compute the following MLE under the Gaussian error model $u_{i,j}\sim N(\widetilde{h}_j(d_p(x_i,y_i)),\sigma^2)$ on the GLM-PCs, where $\widetilde{\mathbf{h}}=(\widetilde{h}_1,\dots,\widetilde{h}_{2P}):\mathbb{R}\to\mathbb{R}^{2P}$ is a 1-D GLM-PC function. That is, we solve

$$\underset{\substack{w_p:\mathbb{R}^2\to\{0,1\}\text{ s.t. }\sum_{p=1}^P w_p(x,y)=1\forall(x,y)\in T\\ d_p(x,y)\in C^1(\mathbb{R}^2,\mathbb{R})\\ \widetilde{h}_{p,j}(z)\in\mathcal{L}(\mathbb{R},\mathbb{R})}}{\operatorname{argmax}} \sum_{j=1}^{2P}\sum_{i=1}^{N}\log\mathbb{P}\left(u_{i,j}\;\middle|\;\sum_{p=1}^{P}\widetilde{h}_{p,j}(d_p(x_i,y_i))\cdot w_p(x_i,y_i)\right). \tag{11}$$

Solving (11) yields estimated isodepth functions $\widehat{d}_p$ and spatial domain assignment functions $\widehat{w}_p$. We then estimate the 1-D gene expression functions $h_{p,g}$ for each gene $g=1,\dots,G$ by solving (11) fixing the isodepth functions $\widehat{d}_p$ and spatial domain assignment functions $\widehat{w}_p$, i.e.

$$\underset{h_{p,g}(z)\in\mathcal{L}(\mathbb{R},\mathbb{R})}{\operatorname{argmax}} \sum_{g=1}^{G}\sum_{i=1}^{N}\log\mathbb{P}\left(u_{i,j}\;\middle|\;\sum_{p=1}^{P}h_{p,g}(\widehat{d}_p(x_i,y_i))\cdot\widehat{w}_p(x_i,y_i)\right). \tag{12}$$

We solve (12) using Poisson regression for each individual gene $g=1,\dots,G$ and spatial domain $p=1,\dots,P$. This is equivalent to solving GP Poisson regression problems. See Ma et al. (2022); Chitra et al. (2025) for details.

Cell type-specific gradients

Briefly, we write the 1-D gene expression function $h_{p,g}(z)$ as $h_{p,g}(z) = \sum_{c=1}^{C} h_{c,p,g}(z) \cdot u_c(z)$ as a sum over cell types $c = 1, \ldots, C$, where $u_c(z)$ is the proportion of cell type c at isodepth z and $h_{c,p,g}(z)$ is the *cell type c-specific* 1-D expression function. Given cell type annotations $\ell_{i,c} \in \{0, 1\}$, where $\ell_{i,c} = 1$ if spatial location i contains cell type c and $\ell_{i,c} = 0$ otherwise, we learn a (linear) cell type-specific 1-D expression function $h_{c,p,g}(z) = \beta_{c,p,g} \cdot d_p(x, y) + \alpha_{c,p,g}$ for each cell type c, across all genes g with an expression gradient in domain R_p as defined above.

A large absolute value of the *cell type-specific* slope $\beta_{c,p,g}$ for any cell type c describes an expression gradient in gene g expression in domain R_p that is specific to cell type c, and thus is not driven by a gradient in cell type c proportion, while a small value of the slope $\beta_{c,p,g}$ for all cell types c indicates that the expression gradient is driven by a gradient in cell type proportion. See Chitra et al. (2025) for specific details.

Simulation

We first evaluated GASTON-Mix on simulated SRT data. We compared GASTON-Mix to four recent methods for analyzing SRT data: BANKSY (Singhal et al., 2024), CellCharter (Varrone et al., 2024), GraphST (Long et al., 2023), and GASTON (Chitra et al., 2025). BANKSY, CellCharter, and GraphST are methods for spatial domain identification which do not explicitly model gene expression gradients within domains, while GASTON is primarily designed for identifying spatial gradients and makes restrictive global assumptions on tissue geometry.

We simulated SRT data $(\mathbf{S}, \mathbf{A})$ on a rectangular tissue T with $N = 10,000$ spatial locations arranged in a 100×100 grid. The tissue T consists of $P = 2$ spatial domains R_1, R_2 arranged in a "checkerboard" pattern (Figure 3A). The domain-1 isodepth function $d_1(x, y)$ (resp. domain-2 isodepth $d_2(x, y)$) is the distance from the nearest left edge (resp. bottom edge) of a square in the checkerboard (Figure 3B). Jackson et al. (2024) previously showed that many existing SRT methods, including GASTON and BANKSY, could not accurately model a checkerboard arrangement of domains. We simulate the expression of $G = 3G'$ genes using three classes of expression functions $f_g(x, y)$ that satisfy the assumptions of Theorem 1: (1) a linear function $f_g(x, y) = \alpha_{1,g} d_1(x, y) + \beta_{1,g}$ of only the domain-1 isodepth function $d_1(x, y)$; (2) a linear function $f_g(x, y) = \alpha_{2,g} d_2(x, y) + \beta_{2,g}$ of only the domain-2 isodepth function $d_2(x, y)$; and (3) a linear function $f_g(x, y) = (\alpha_{1,g} d_1(x, y) + \beta_{1,g}) \cdot w_1(x, y) + (\alpha_{2,g} d_2(x, y) + \beta_{2,g}) \cdot w_2(x, y)$ of both domain-specific isodepths $d_1(x, y), d_2(x, y)$. We draw the gene expression function parameters $\alpha_{p,g}, \beta_{p,g} \sim U(-1, 1)$ uniformly at random for each gene $g = 1, \ldots, G$ and domains $p = 1, 2$ (Figure S1A). We draw gene expression values $a_{i,g} \overset{\text{i.i.d.}}{\sim} N(f_g(x_i, y_i), 1)$ from a normal distribution centered at $f_g(x, y)$ for $G' = 5$ genes from each class (Figure S1B). When running all methods, we omit the pre-processing steps (e.g. PCA) as the gene expression values are already low-dimensional and normally distributed, allowing us to compare the different algorithms.

We find that GASTON-Mix achieves much higher accuracy in spatial domain identification, measured in terms of the adjusted Rand index (ARI), compared to BANKSY, CellCharter, GraphST, or GASTON (GASTON-Mix mean ARI ≈ 0.85 versus mean ARI < 0.70 for other methods; Figure 3C). The lower ARI of BANKSY, GraphST, and CellCharter is likely because these methods do not explicitly model gene expression gradients, while GASTON's poor performance is because the simulated data does not satisfy GASTON's global isodepth assumption (Theorem 1). We note that while BANKSY claims to model *"gradients in gene expression in [cellular] neighborhoods"* (Singhal et al., 2024), the BANKSY domains have the lowest ARI which suggests that BANKSY is not accurately modeling gradients. In contrast, GASTON-Mix's local model of tissue geometry and expression gradients allows for accurate inference of spatial domains.

We next evaluated the ability of GASTON-Mix and other methods to learn spatial gradients. We specifically evaluated how well each method learns the domain-specific isodepth functions $d_i(x, y)$, which provide coordinates for quantifying spatial gradients. We measure the mean Kendall's τ correlation between the true domain-specific isodepth coordinates $d_1(x, y), d_2(x, y)$, and the isodepth(s) learned by GASTON and GASTON-Mix. While GraphST does not explicitly learn a coordinate for measuring gene expression gradients, GraphST learns a 20-dimensional embedding vector at each spatial location, and we evaluate GraphST using the largest Kendall's τ correlation between each component of the GraphST embedding and the domain-specific isodepth coordinates $d_1(x, y), d_2(x, y)$. We do not compare against BANKSY and CellCharter which only identify spatial domains and do not learn a coordinate within each domain for measuring gradients.

We find (Figure 4A) that GASTON-Mix has larger Kendall's τ correlation ($\tau \approx 0.33$) with the true domain-specific isodepths than GASTON ($\tau \approx 0.27$) or GraphST ($\tau \approx 0.17$). GraphST is unable to learn a spatially smooth embedding (Figure 4B) while the GASTON isodepth function is constant in several of the squares (Figure 4C), likely because there does not exist a continuous isodepth function d that describes the describes the gene expression function $\mathbf{f}(x, y)$ (see Theorem 1). On the other hand, the GASTON-Mix domain-specific isodepths smoothly vary across the domains (Figure 4D), showing the advantages of learning *local* topographic maps.

Evaluating CellCharter initialization

For the MERFISH mouse anterior forebrain analysis, we also ran GASTON-Mix without initializing the gating function using the CellCharter initialization (Methods), instead using the default PyTorch initialization. The resulting spatial domains have an ARI of 0.432 compared to the CCF domain labels, which is larger than the ARI of other methods (ARI ranging from $0.11 - 0.41$) but is less than the ARI of GASTON-Mix with pre-training (0.734). The difference in ARIs is likely because the model loss (i.e. the negative of the log-likelihood in (8)) with the CellCharter initialization is noticeably smaller than the model loss *without* the CellCharter initialization (loss 0.89 without initialization versus loss 0.87 with initialization). Thus, the CellCharter initialization allows the MoE model to converge to a better local optimum.

Spatial gradients in lateral septum

GASTON-Mix identifies biologically relevant spatial gradients in the lateral septum (LS) domain (Figure 5A). Spatial gradients in the LS have previously been observed in other spatial imaging data and are hypothesized to be involved in complex social behaviors including reward processing and motor learning (Chen et al., 2024; Risold and Swanson, 1997; Reid et al., 2024). In particular, GASTON-Mix identifies two genes which were recently observed to be markers of several subtypes of lateral septum neurons in a separate MERFISH lateral septum dataset (Reid et al., 2024), *Ano2* and *Lamp5*, as having large LS-specific gradients (Figure S4C-J). These gradients may be driven by gradients in cell type proportion, e.g. the decreasing gradient in "LSX GABA" cell type proportion (Figure S4B).

Gradients in breast cancer tumor

We applied GASTON-Mix to a 10x Genomics Visium SRT sample of a human breast cancer (invasive ductal carcinoma) where the expression of $36,601$ genes was measured in $3,798$ spots (Xu et al., 2022). Physical and biochemical gradients within a tumor have been previously shown to promote cancer progression and metastasis (Oudin and Weaver, 2016). GASTON – the current state-of-the-art method for identifying spatial gradients from SRT data – requires the spatial domains of a tissue to satisfy restrictive geometric assumptions (Chitra et al., 2025). However, the pathologist annotations for this cancer sample (Figure 6A) suggest that its spatial domains have a complex geometry that does not satisfy the GASTON assumptions. Thus, we hypothesized that the more flexible model of GASTON-Mix would allow us to identify spatial domains and gradients inside this tissue.

We first compared the $P = 8$ domains identified by GASTON and GASTON-Mix. We observe that the GASTON spatial domains (Figure 6B) have noticeably less agreement with the pathologist annotations than the GASTON-Mix domains (Figure 6C), both visually and quantitatively (GASTON ARI ≈ 0.10 versus GASTON-Mix ARI ≈ 0.19 compared to pathologist annotations). This provides evidence that this data does not satisfy the GASTON assumptions, and demonstrates the improved flexibility of GASTON-Mix in modeling arbitrary arrangements of spatial domains compared to GASTON.

We next used GASTON-Mix to identify *shared* and *domain-specific* spatial gradients across the different domains of the cancer sample. Specifically, we identified the set of genes with domain p-specific expression gradients across domains $p = 1, \dots, P$ (Methods) and subsequently examined the enrichment of these genes in known cancer hallmark gene sets (Figure 6D). We observe that some hallmark sets are enriched across many domains, i.e. they are *shared* across domains, while other sets are enriched in specific domains, i.e. they are *domain-specific*. For example, genes involved in TNF-α signaling such as *CCL2* (Ho et al., 2008) exhibit spatial gradients specifically in invasive domain R_2 (Figure 6E) and genes involved in hypoxia such as the hypoxia-inducible gene *ALDOA* (Niu et al., 2021) exhibit spatial gradients specifically in invasive domain R_3 (Figure 6F). On the other hand, gradients of genes involved in the epithelial-mesenchymal transition (EMT), such as *CDH11* (Schneider et al., 2012), are present across multiple domains including invasive domains R_3 and R_7 (Figure 6G). We note that the spatial gradients identified by GASTON-Mix would be challenging to identify using existing SRT methods which do not learn coordinates that smoothly vary within domains (e.g. GraphST embeddings in Figure S5).

Thus, GASTON-Mix reveals spatial gradient patterns that characterize the different domains of a cancer sample.

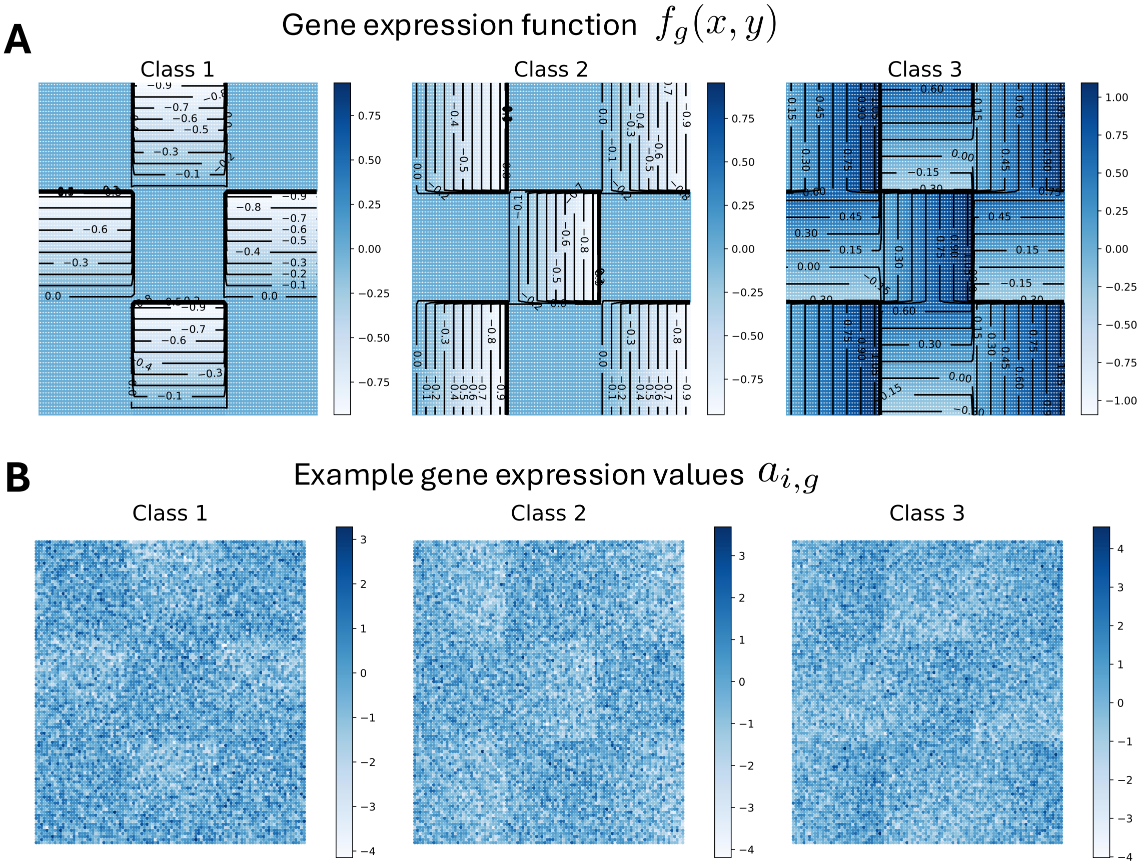

Fig. S1. (A) Three classes of gene expression functions $f_g(x, y)$. **(B)** Three examples of simulated gene expression $a_{i,g}$ from each of the classes of gene expression functions shown in (A).

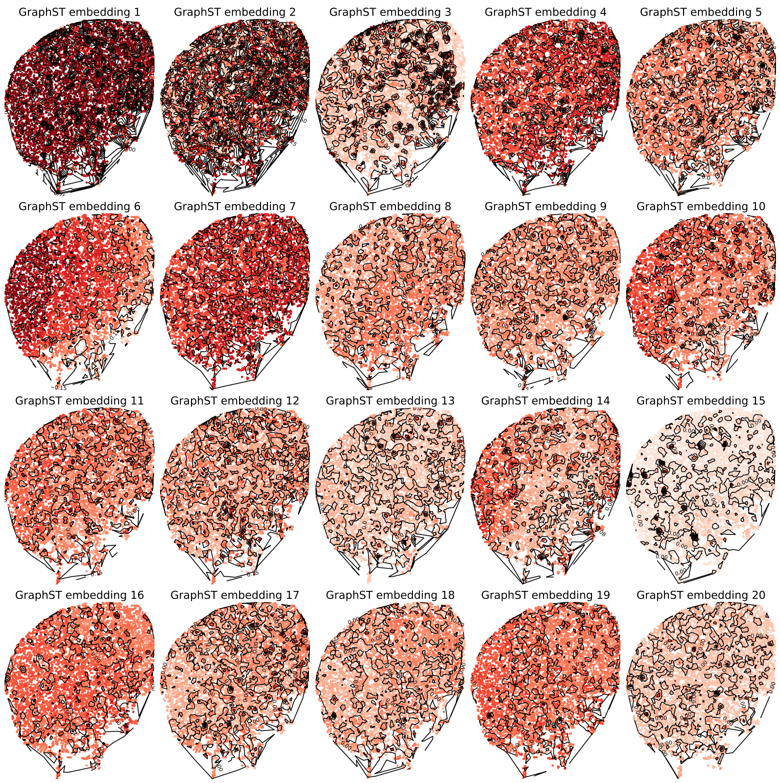

Fig. S2. GraphST embedding components for GASTON-Mix-identified CP domain in MERFISH mouse forebrain. Curves connect spots of equal embedding value.

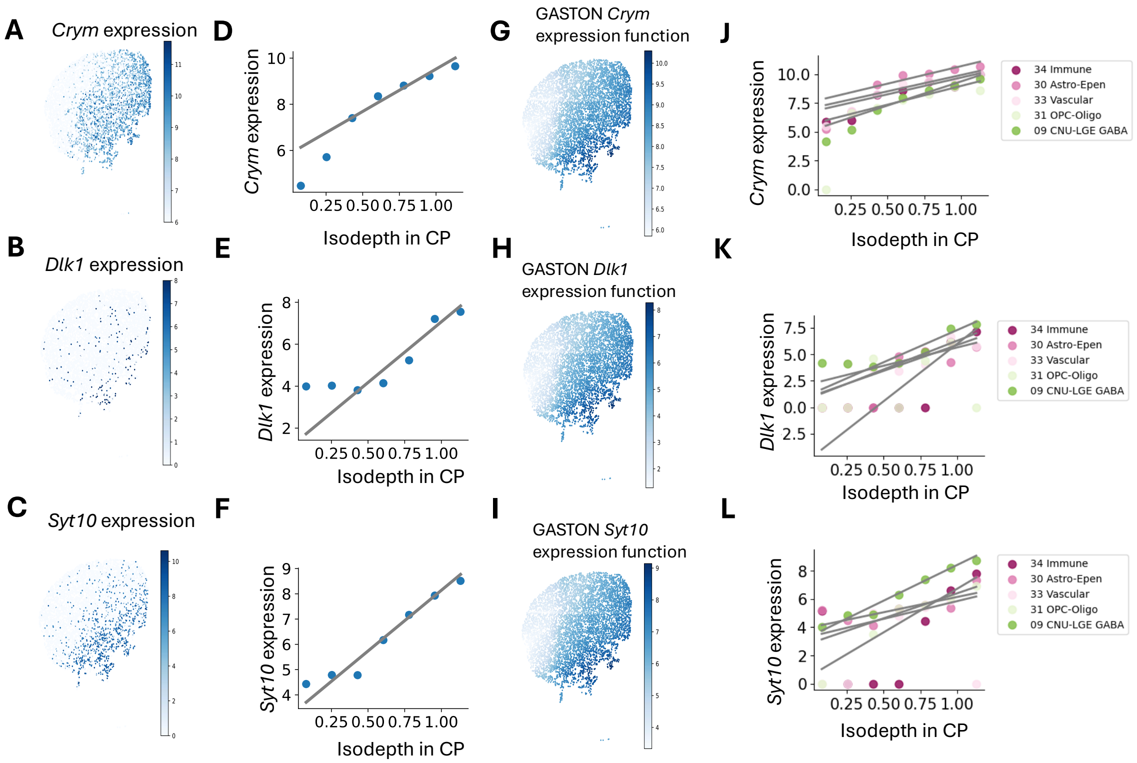

Fig. S3. (A-C) Gene expression in CP domain for (A) *Crym*, (B) *Dlk1*, and (C) *Syt10*. **(D-F)** Isodepth in CP versus expression for (D) *Crym*, (E) *Dlk1*, and (F) *Syt10*, three candidate markers for the dorsolateral-ventromedial striatum gradient Märtin et al. (2019). **(G-I)** GASTON CP-specific expression function for (G) *Crym*, (H) *Dlk1*, and (I) *Syt10*. **(J-L)** Isodepth in CP versus cell type-specific expression functions for (J) *Crym*, (K) *Dlk1*, and (L) *Syt10*.

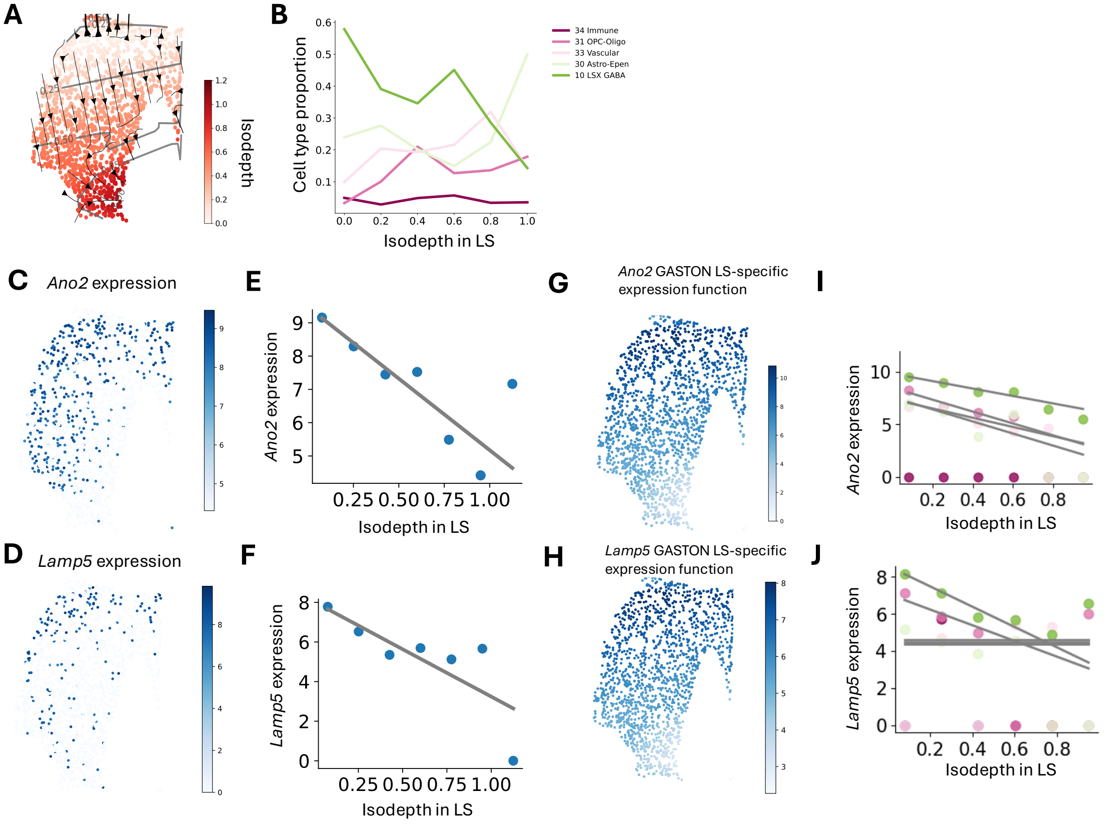

Fig. S4. (A) Isodepth in lateral septum (LS) domain labeled in Figure 5B. **(B)** Cell type proportion versus GASTON-Mix-inferred isodepth in LS. **(C-J)** Same figures as in Figure S3 for genes *Ano2* and *Lamp5*.

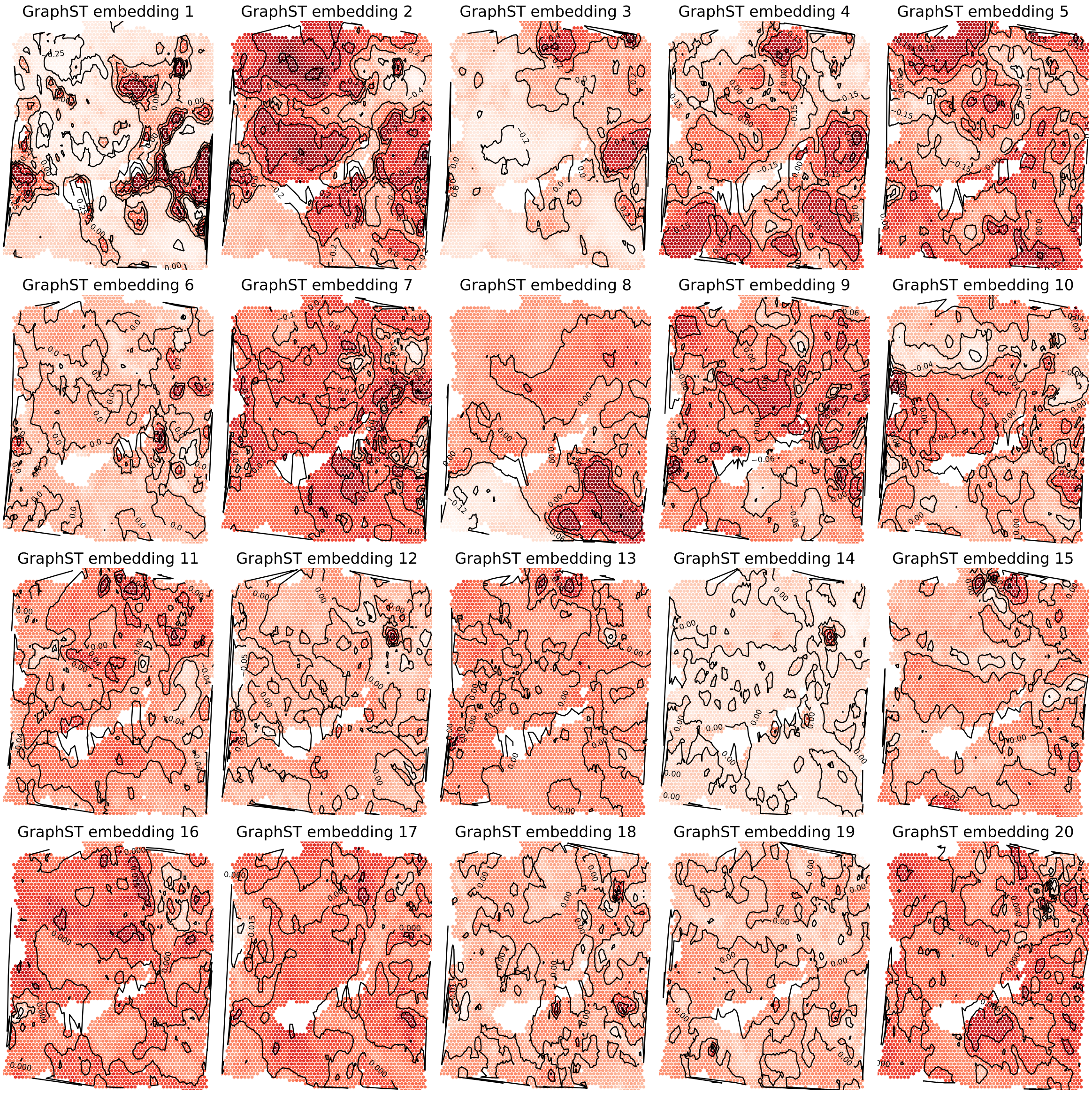

Fig. S5. GraphST embedding components for 10x Visium breast cancer sample. Curves connect spots of equal embedding value.
